# A Preliminary Study of the Proportion of Different Chronic Headache Types and Their Effect on Quality of Life at a Tertiary Care Hospital in North India

**DOI:** 10.7759/cureus.84224

**Published:** 2025-05-16

**Authors:** Pragati Dwivedi, Kiran Jakhar, Sanjay Badesara, Ruchi Verma, Nidhi Dixit, Shailly Raj

**Affiliations:** 1 Department of Psychiatry, Government Institute of Medical Sciences, Greater Noida, IND; 2 Department of Obstetrics and Gynaecology, Venkateshwara Institute of Medical Sciences, Gajraula, IND; 3 Department of Obstetrics and Gynaecology, Government Institute of Medical Sciences, Greater Noida, IND; 4 Department of Ophthalmology, Government Institute of Medical Sciences, Greater Noida, IND

**Keywords:** cephalgias, headache, migraine, quality of life, tension-type headache

## Abstract

Introduction

Globally, chronic headaches are one of the most common health problems affecting the productive age group and an individual's quality of life.

Aims

This study aims to find the proportion of different types of chronic headaches and their impact on an individual's quality of life.

Methods

This cross-sectional observational study included patients with chronic headaches (15 or more episodes per month for at least three months, as per the International Headache Society) in the outpatient department of a tertiary care hospital. The patients were evaluated on a semi-structured proforma, MINI (Mini-International Neuropsychiatric Interview) version 7.0.0, ICHD-3 (International Classification of Headache Disorders-Third Edition), and the Chronic Headache Quality of Life Questionnaire (CHQLQ).

Results

A total of 100 subjects with chronic headaches were recruited in the study, which comprised 4.45% of the total number of psychiatry outpatients over the given period. Among the subjects of chronic headache, tension-type headache (TTH) was highest at 61 (61%), followed by migraine at 25 (25%). Out of these, approximately two-thirds were females with a mean age of 36.13±12.93 years. The data analysis frequency and duration of the headache episode were statistically significant, with a p-value of <0.01. All types of headaches negatively affected most of the parameters of quality of life, but they are not statistically significant except for two, i.e., household chores and feeling of burden on others, where the p-value was 0.03 and 0.04, respectively.

Conclusion

The proportion of patients presenting to the Psychiatry Outpatient Department was significant, emphasizing that TTH is a highly prevalent entity causing deterioration in quality of life.

## Introduction

One of the most common health problems is headache, which affects around 90% of people at least once in their lifetime [1]. As per the data of the Global Burden of Diseases, Injuries, and Risk Factors (GBD) studies, the major emerging public health concern is headache [2]. International Classification of Headache Disorders-Third Edition (ICHD-3) classifies headaches into the major categories of primary and secondary headaches. The major bulk of the primary headache is due to tension-type headache (TTH) [3], migraine [4], and medication overuse. Of all GBD causes of disease, TTH was the third most prevalent, while migraine ranked sixth [2].

The most common age for primary headache is the end of puberty and the beginning of the fifth decade of life, thus affecting the major productive years of an individual [5]. Headache affects the functioning of an individual at the workplace, school, household, financial aspects, quality of life, and overall well-being [6]. As per the studies, migraine ranked second globally among the 10 most disabling disorders [2] and is one of the most restrictive disorders. On the other hand, TTH is milder in intensity, which causes impairment of day-to-day activities, resulting in disability [7].

A study by Shapiro et al. in 2024 revealed that the burden of migraine affects the optimal functioning of an individual's ability to fulfill life goals. Those individuals with migraine may also experience stigma associated with the condition [8]. However, the literature concerning other types of primary headache remains limited.

There has been no hospital-based study from Northern India that focuses on the epidemiology of different types of headaches and their relation with quality of life. The individuals suffering from headaches report to departments like Internal Medicine, Ophthalmology, Neurology, Psychiatry, or the Pain clinic (if it exists). The aim of this cross-sectional observational study was to determine the proportion of chronic headaches in the Outpatient Psychiatry Department and their impact on quality of life in the Western part of Uttar Pradesh, a previously unexplored area for the status of various types of headache.

## Materials and methods

Approval from the Government Institute of Medical Sciences (GIMS) Institutional Ethical Committee was obtained prior to the start of the study (reference no. GIMS/IEC/HR/2022/26). An observational study with consecutive sampling was done at a tertiary care center.

All male and female patients presenting to the psychiatric outpatient department of the tertiary care hospital with chronic headaches who were willing to participate and agreed to give written informed consent were included in the study. Chronic headaches consist of 15 or more episodes per month for at least three months, as per the International Headache Society. At the start of the study, the subjects were informed briefly about its purpose, advantages, and disadvantages, and that they would not receive any compensation. Participation by accompanying relatives will be welcomed to obtain as much additional information as possible. Inclusion criteria were age above 18 years, both genders, from the outpatient department with the diagnosis of headache by ICHD-3 [9], and giving written informed consent. The exclusion criteria were patients having chronic medical, psychiatric/substance dependence, or neurological illness, having refractive error, ear pathology/tinnitus, or intellectual disability.

The sample consisted of 100 patients with chronic headaches, with consecutive sampling. All patients presenting to the outpatient department of psychiatry at the tertiary care hospital comprised the study population. After giving written informed consent, the subjects were recruited for the study. Subsequently, the subjects were assessed using a semi-structured proforma designed to collect information on sociodemographic and clinical variables of the study population. In order to maintain the purity of the sample, the MINI (Mini-International Neuropsychiatric Interview) version 7.0.0 [10] was applied to exclude the subjects who had any other psychiatric diagnosis. Following this, all the subjects were categorized into different types of headaches, such as TTH or migraine, as per ICHD-3 [9], and their quality of life was ascertained using the Chronic Headache Quality of Life Questionnaire (CHQLQ) [11]. The scores obtained on different measures were arranged and entered into MS Excel sheets (Microsoft Corporation, Redmond, Washington), and then the data were analyzed.

International Classification of Headache Disorders (ICHD-3)

The ICHD was designed by the Classification Committee of the International Headache Society. ICHD-3, published as the first issue of Cephalalgia in 2018, is an algorithmic system to define and classify all known headache disorders. It consists of four parts: primary headaches, secondary headaches, neuropathies and facial pains, and other headaches and appendices [9].

Semi-structured proforma

The semi-structured proforma consisted of two parts, which included sociodemographic information and details containing information such as ID number, age, gender, religion, education, marital status, occupation, socioeconomic status, contact details, and clinical details (age, duration of illness, frequency of headache, duration of headache, and severity).

Mini-International Neuropsychiatric Interview (MINI) version 7.0.0

This questionnaire is designed to evaluate the presence of psychiatric disorders in a population. It has a reliability and validity similar to the Structured Clinical Interview for DSM-IV (SCID) and the Composite International Diagnostic Interview (CIDI). It is divided into many sections, each of which is depicted by a letter. Each letter stands for a diagnostic category. Answers are in a yes or no format. It is intended to be used as a tool to facilitate accurate data collection and processing of symptoms elicited by trained personnel. It is used widely in psychiatric literature. It can be applied in a very short period of time, estimated to be a median of 15 minutes. It will be used to screen controls prior to inclusion in the study. It is available free of charge and does not require any permission to be used in a research project.

Chronic Headache Quality of Life Questionnaire (CHQLQ)

This questionnaire is designed to measure the impact of chronic headaches on a person's overall quality of life, focusing on functional aspects. The CHQLQ is a 14-item questionnaire that assesses the functional aspects of headache-related quality of life, producing three domain scores (role prevention, role restriction, and emotional function).

IBM SPSS Statistics for Windows, Version 21 (Released 2012; IBM Corp., Armonk, New York), was used for data analysis. Frequency distribution in terms of mean and standard deviation, proportions, and percentages was carried out for sociodemographic details. The statistical procedure used included the chi-square test. A p-value less than 0.05 was considered statistically significant.

## Results

A total of 100 subjects diagnosed with chronic headache by the International Headache Society were recruited for the study. These subjects were recruited over a seven-month duration, where a total of 2245 patients reported to the outpatient department of psychiatry, which means the proportion of chronic headache is 4.45% in the department.

Out of 100 recruited subjects, approximately two-thirds were females, while one-third were males. The mean age of male patients was 33.19±9.49 years, while the mean age of females was 36.13±12.93 years. The majority of the subjects followed Hinduism, 92 (92%), while approximately eight (8%) of the subjects followed other religions like Islam. With respect to education, 82 (82%) were literate, and 18 (18%) were illiterate. As per the marital status, 74 (74%) were married. Less than half were either unemployed, housewives, or students. The majority of the subjects with headaches were from middle socioeconomic backgrounds, while upper and lower statuses had a share of 31 (31%) and 19 (19%), respectively (Table 1).

**Table 1:** Sociodemographic profile of the subjects

Variable		N (%)
Gender	Male	68 (68%)
	Female	32 (32%)
Religion	Hindu	92 (92%)
	Muslim	8 (8%)
Education	Literate	82 (82%)
	Illiterate	18 (18%)
Marital status	Married	74 (74%)
	Never married	26 (26%)
Occupation	Unemployed/housewife/students	45 (45%)
	Skilled worker/professional	31 (31%)
	Unskilled/farmer	24 (24%)
Socioeconomic status	Upper	31 (31%)
	Middle	50 (50%)
	Lower	19 (19%)

In the current study, the most common type of headache was TTH, followed by migraine and mixed headache. Although there was no significant difference in various types of headaches as per gender profile, there was a female predominance in each type (Table 2).

**Table 2:** Distribution of different types of chronic headache TTH: tension-type headache; Mixed: TTH and migraine

Type of Chronic Headache	Total (%)	Male (%)	Female	P-value
TTH	61 (61%)	15 (15%)	46 (46%)	0.1
Migraine	25 (25%)	10 (10%)	15 (15%)	
Mixed	14 (14%)	7 (7%)	7 (7%)	

The mean age group of the subjects with chronic headache was 35.2±11.9 years for all groups, while the total duration of illness was 3.8±4.2 years. The total mean headache frequency was 8.22±6.14, which was significant, with TTH having the highest frequency among all types. While the total mean duration of headache episodes was 7.97±6.3 hours, the highest episode duration was for migraine, followed by mixed-type headache and TTH, respectively (Table 3).

**Table 3:** Clinical profile of the subjects *The data was not normally distributed with right-sided skewness, so the standard deviation (SD) is greater than the mean. TTH: tension-type headache

Characteristics	Total	TTH	Migraine	Mixed	P-value
Age	35.2±11.9	36±12.4	35.08±11.8	31.8±10.5	0.61
Total duration of headache*	3.8±4.2	3.6±4.3	4.2±4.28	4.1±4.1	0.53
Frequency of headache	8.22±6.14	9.62±6.23	5.0±3.1	7.86±7.8	<0.01
Duration of the headache episode	7.97±6.3	6.20±4.19	11.40±7.93	9.57±8.28	<0.01
Severity of headache	2.0±0.75	2.02±0.78	1.96±0.68	2.0±0.78	0.76

Multiple domains of quality of life, including interference with family, interference with leisure time, and often feeling fed up and frustrated, were negatively affected by the different types of headache, but these effects were not statistically significant. However, the two parameters of needing help to do household chores and feeling of burden on others were statistically significant, with p-values of 0.03 and 0.04, respectively (Table 4).

**Table 4:** Relationship between chronic headache type and quality of life TTH: tension-type headache

Domain	TTH	Migraine	Mixed	Average Mean	P-value
Dealt with family	2.39±0.94	2.04±0.79	2.07±0.91	2.26±0.92	0.08
Leisure time	2.75±.87	2.40±.91	2.75±1.0	2.61±.91	0.07
Difficulty daily	2.51±.85	2.52±.92	2.50±1.1	2.51±.89	0.96
As much work	2.46±.87	2.32±0.9	2.50±1.02	2.43±.86	0.54
Concentrate	2.69±.87	2.48±.92	2.36±.87	2.59±.91	0.26
Too tired	2.57±.71	2.36±.86	2.57±1.02	2.52±.81	0.30
Energetic days	2.43±.76	2.20±1.04	2.21±.96	2.34±.87	0.24
Cancel work	2.16±.95	2.08±.90	2.21±0.97	2.15±.94	0.75
Household chores	2.38±0.8	1.96±1.02	1.93±0.83	2.21±.88	0.03
Stop work	2.30±.96	2.02±1.02	1.93±1.0	2.18±.98	0.21
Social activity	2.49±1.01	2.12±1.09	2.43±1.16	2.39±1.05	0.15
Fed up	2.89±.84	2.52±.82	2.50±1.09	2.74±.88	0.06
Feeling of a burden on others	2.16±.93	1.68±0.85	2.14±1.18	2.04±.96	0.04
Letting down	2.05±1.02	1.84±1.10	2.07±1.2	2.00±1.06	0.45

## Discussion

According to the available literature, chronic headaches are among the most common health problems in the productive age group. Primary headaches like TTH and migraine are most prevalent [1,3,4]. These types of headaches take a huge toll on an individual's daily functioning, leading to deterioration in the quality of life. The current study evaluates the proportion of headaches with their subtyping and their impact on the quality of life.

The current study recruited 100 subjects with chronic headaches, approximately 4.45% of the hospital-based data. However, population-based studies reported as high as 46% with active headache disorder [12]. The data included only patients who reported to the outpatient department of psychiatry and not all other departments from where the patients would be seeking treatment. The huge difference could also be attributed to a hospital-based study, with only severe forms reporting to the hospital and the stigma attached to it.

In the current study, TTH was the most common entity, accounting for approximately 61%, followed by migraine, which was 25%, and the least common was mixed type, which included subjects suffering from both TTH and migraine, accounting for around 14%. The finding of the current study correlates with the available literature, which also reports 42% and 11% of TTH and migraine, respectively [12]. The results are also in concordance with GBD studies, which also state that TTH is much more common than migraine [2].

In the current study, the mean age of experiencing headaches was slightly higher in females (36 years) than in males (33 years). However, the age group that was reported to have headaches from a study in South India was 38 years [13], and as per GBD studies, it was 15 to 49 years [2]. This variation in age could be attributed to the different sample sizes, focusing only on medical students, schools, adults, hospital-based studies, or population-based studies. In the current study, approximately two-thirds were females, while one-third were males, which is in concordance with the available literature [1,2,6,13-15].

In the current study, approximately 80% of the literate subjects suffered from various types of headaches, which is in concordance with the literature [6]. Additionally, all types of headaches negatively affected quality of life and were statistically significant in the domains of doing household chores and feeling a burden on others. In a study by Shapiro et al., interictal burden, migraine-specific quality of life, and increased migraine-related stigma were associated with increased disease burden across all monthly headache day categories [8]. In a study by Acikgoz et al., all quality-of-life factors were worse in those with a longer duration of headache [6].

The current study is a preliminary study to assess the proportion of headaches, their subtyping, and their impact on quality of life in a hospital-based scenario in Western Uttar Pradesh. The study underscores the importance of assessing and addressing headaches as a concerning health issue. The sample size of the study is also relatively small.

The limitation of this study is that it is a preliminary study with a small sample size. It includes data from one of the departments of a tertiary care center. Therefore, the generalization of the results is not advisable. The prevalence and one-year incidence of headaches in the general population may be much larger. Moreover, the study excluded all psychiatric diagnoses with the aim of reducing any confounding factors. However, it is possible that the study excluded significant comorbidities related to headaches.

## Conclusions

The current study reemphasized the fact that among all types of primary headache, TTH is the most common entity, followed by migraine, with a higher female preponderance. It also emphasized that the most common age group in the literate population is between 30 and 40 years old and older. All types of headaches worsen all the dimensions of quality of life, especially disabling the individual from doing household chores and placing a burden on others. Hence, the finding of this study encourages more scientific research in the field of headaches in the Indian population, along with specific and timely interventions for effective treatment to improve quality of life, as it primarily affects the productive age of the population.
